# Physicians' Attitudes Towards Treating Patients in the Context of COVID-19 Pandemic in Pakistan

**DOI:** 10.7759/cureus.10331

**Published:** 2020-09-09

**Authors:** Maheen Ayub, Daneyal Arshad, Naheed Maqbool, Mahnoor Zahid, Rabia S Malik, Zuhair Ali Rizvi, Usman Arshad, Saleem Ullah Khan

**Affiliations:** 1 Internal Medicine, Rawalpindi Medical University, Rawalpindi, PAK; 2 Anesthesiology, Shifa International Hospital, Islamabad, PAK; 3 Community Health Sciences, Ziauddin University, Karachi, PAK; 4 Pediatric Surgery, Children's Hospital and Institute of Child Health, Lahore, PAK

**Keywords:** covid-19, personal protective equipment, reluctance, public health care, mental health

## Abstract

Background and objectives

Severe acute respiratory syndrome coronavirus 2 (SARS-CoV-2) has been the cause of a worldwide outbreak of respiratory illness, which has been declared as coronavirus disease 2019 (COVID-19) pandemic by the World Health Organization (WHO). The outbreak has posed a huge challenge to countries around the world and has resulted in a global lockdown. The pandemic has especially overburdened the healthcare sector, resulting in a shortage of personnel and equipment. Along with many other manifestations, it has resulted in stress and anxiety for the physicians as well. Furthermore, many healthcare workers have been reluctant in treating COVID-19 patients. This study aimed to explore the concerns of physicians in the context of the COVID-19 pandemic and to evaluate the reasons for their reluctance to treat the patients.

Methodology

This descriptive cross-sectional study included 235 physicians from seven hospitals of Pakistan who were actively working amid the COVID-19 pandemic. Data were collected from March 1, 2020, to May 30, 2020, using a structured online questionnaire. Participants were approached via non-probability convenient sampling. Two hundred and eight respondents were included in the data analysis. SPSS Statistics version 23.0 (IBM Corp., Armonk, NY) was used for data entry and analysis.

Results

A striking 83.7% (n=174) of the respondents expressed their reluctance to treat patients with COVID-19. Concerns they raised included one or more of the following four reasons; lack of proper personal protective equipment (PPE), fear of self-infection, excessive workload, and fear of transmitting the infection to their family members. Of note, 92% (n=161) of the respondents reported a lack of PPE while 74.1% (n=129) reported fear of transmitting the infection to their family members as reasons for their reluctance. The vast majority of the participants reported the need for psychological training to treat the patients' anxiety (95.2%, n=198). Many participants were afraid that their own anxiety might be affecting the quality of care patients were receiving (67.3%, n=140). Hence, most of the participants reported that psychological counseling should be provided (93.3%, n=194). Participants with family members older than 60 years were found to be reluctant to treat patients due to the risk of transmitting the infection to them (69.7%, n=145, p=0.001). Therefore, a major proportion of the participants (96.2%, n=200) felt that the hospitals should provide a place for them to rest and temporarily isolate themselves to avoid coming into contact with their family members.

Conclusions

We conclude that a major proportion of physicians is reluctant to treat their patients due to multiple factors. The grave situation of the pandemic has taken a toll on their mental health, which could be affecting the quality of care that the patients receive. Their concerns should be addressed to not only provide them with support and improve their working environment but also to ensure that they are fully equipped to provide state-of-the-art care to the patients in these grave times.

## Introduction

The World Health Organization's (WHO) country office in China received the first notification about a pneumonia of unknown origin in Wuhan, Hubei province on December 31, 2019. On January 2, 2020, 41 patients admitted to a hospital in Wuhan, China, were found positive for the novel coronavirus, later termed as severe acute respiratory syndrome coronavirus-2 (SARS-CoV-2). The Director-General of WHO declared the novel coronavirus outbreak a public health emergency of international concern on January 30, 2020. On February 26, 2020, Pakistan confirmed its first two cases of coronavirus in Karachi and Islamabad. By March 13, there were 737 deaths and 34,336 cases reported in Pakistan [1].

The disease caused by SARS-CoV-2 was termed coronavirus disease 2019 (COVID-19) [2]. It is an infectious disease manifesting as a respiratory illness that can range from common cold to severe pneumonia and can be fatal in some cases. It is transmitted from one person to another through respiratory droplets. Transmission by indirect contact with surfaces has also been reported [3]. The symptoms can range from mild fever, cough, and shortness of breath to severe symptoms like pneumonia and kidney failure, which can lead to fatal outcomes [2]. Frequent washing of hands, wearing a mask, and social distancing have proven to limit the risk of exposure.

With the emergence of this pandemic, the number of patients presenting to the hospitals has drastically increased. The workload for healthcare workers dealing with COVID-19 patients has also increased dramatically, as confirmed by a study conducted in Iran [4]. In addition to being a huge challenge to the physical well-being of the society, the COVID-19 pandemic is seriously affecting the mental health of not only the general public but also the healthcare providers. The psychological toll that managing the patients with COVID-19 has on the mental health of physicians and nurses has been unprecedented. The care-givers have to often decide on the allocation of the limited resources to the patients that need it the most. A British Broadcasting Corporation (BBC) article has rightly described the situation as "the young doctors being asked to play god" [5]. Furthermore, many healthcare workers are reluctant to come to hospitals and treat COVID-19 patients, as demonstrated by a report by the Columbia Broadcasting System (CBS) News [6]. A significant number of healthcare workers have themselves contracted the disease and many have also died due to the disease.

The psychological impacts have been the result of overwhelming work as well as an inherent panic. They can impair the quality of care provided to the patients which, in this chaotic situation, would be disastrous. Therefore, we aimed to report the concerns of physicians amid the pandemic and to assess the reasons for their reluctance to treat COVID-19 patients. We sought to provide a comprehensive overview, in order to highlight the possible ways to ameliorate the concerns of physicians in the context of the ongoing pandemic.

## Materials and methods

This cross-sectional observational study was conducted among physicians from seven hospitals of Punjab, which included Holy Family Hospital, Benazir Bhutto Hospital, District Headquarters Hospital, Maroof International Hospital, Shifa International Hospital, Nishtar Hospital, and Basic Health Unit of District Kasur. A total of 235 physicians, who were actively working in these hospitals during the pandemic, were approached via convenient sampling. Data were collected using a structured questionnaire from March 1, 2020, to May 30, 2020. Doctors who were not involved in screening, diagnosing, and managing COVID-19 patients were excluded from the study.

After getting approval from the relevant ethical review board, the participants were approached and explained the purpose and importance of this study. Consent was obtained and confidentiality was ensured throughout the study. The participants were also informed that they could opt out of the study at any time.

Data were analyzed using SPSS Statistics version 23.0 (IBM Corp., Armonk, NY). Fourteen participants had missing data and were excluded from the analysis. Six participants withdrew consent. Data analysis included 208 participants with complete data. Descriptive statistics were obtained. Means and standard deviations were reported for normally distributed variables. Independent samples t-test and chi-square tests were used wherever applicable.

## Results

Data analysis included 208 respondents, among which 33.7% (n=70) were males and 66.3% (n=138) were females. The mean age of the respondents was 25.44 ±2.34 years. The majority of the participants consisted of house officers (66.8%, n=139) and medical officers (19.2%, n=40) (Table 1). Of note, 68% (n=143) of the participants had no prior experience of working during an epidemic. Only 10.6% (n=22) of the respondents reported undergoing special training to deal with and treat COVID-19 patients.

**Table 1:** Demographic characteristics of the participants

Parameters	Categories	Frequencies	P-value
Gender	Male	70 (33.7%)	
	Female	138 (66.3%)	
Mean age	25.44 ±2.34 years		
	Male	25.40 ±1.76 years	0.854
	Female	25.46 ±2.60 years	
Designation	House officers	139 (66.8%)	
	Medical officers	40 (19.2%)	
	Postgraduates	14 (6.7%)	
	Senior registrars	7 (3.4%)	
	Assistant professors	7 (3.4%)	
	Associate professors	1 (3.4%)	
Mean working hours (per week)	73.38 ±42.49 hours		
	Male	72.44 ±33.49 hours	0.801
	Female	73.86 ±46.49 hours	

A staggering 83.7% (n=174) of the participants expressed their reluctance to treat COVID-19 patients. Reasons for their reluctance were one or more of the following four hazardous factors: lack of proper personal protective equipment (PPE), fear of self-infection, excessive work burden, and fear of transmitting Infection to their family members; 92% (n=161) reported a lack of proper PPE while 74.1% (n=129) respondents expressed their fear of transmitting the infection to their families and loved ones as reasons for their reluctance.

Among the participants, 17% (n=36) reported simultaneous exposure to trauma and stressful conditions, and the majority of these participants were house officers (58.33%, n=21, p=0.26); 34% (n=71) of the participants reported that there was no trend of shift rotation at their hospitals, and 22% (n=46) of the respondents reported living in unfavorable living conditions in the doctor's rooms at the hospitals.

Significantly, 76% (n=160) of the participants reported that they were overwhelmed with their workload (p<0.001); 88% of respondents (n=185) reported that they did not know how to deal with patients who were not willing to be quarantined or were resisting to take proper protective measures. And 95% of respondents (n=198) felt that there should be psychological training to deal with patients who were anxious or panicking. Nearly all participants (99.03%, n=206) reported that staff should be available to deal with uncooperative patients.

Remarkably, 92% of respondents (n=192) reported that they faced a shortage of masks and other protective measures. All participants felt the need for training on how to use protective equipment; 78% of the respondents (n=164) had people older than 60 years in their households. Of note, 96% of the participants (n=200) expressed that the hospital should provide a place where the staff could rest and temporarily isolate themselves from their family to avoid exposing themselves to and infecting their families. Table 2 provides a comprehensive overview of all the questions posed to the participants and their responses.

**Table 2:** Questionnaire responses ^a^Percentage of doctors who were reluctant (n=174) PPE: personal protective equipment

Questions			
1. Have you ever worked in an epidemic before?	Yes	65	31.3%
	No	143	68.8%
2. Are you reluctant to take care of (suspected or diagnosed) corona patients?	Yes	174	83.7%
	No	34	16.3%
3. Did you have training on how to approach and deal with these patients?	Yes	22	10.6%
	No	186	89.4%
4. Do you know how to deal with patients unwilling to be quarantined or to take protective measures?	Yes	23	11.1%
	No	185	88.9%
5. Do you face a shortage of masks and other protective equipment?	Yes	192	92.3%
	No	16	7.7%
6. Are there people older than 60 years at your home that you’re in contact with?	Yes	164	78.8%
	No	44	21.2%
7. Do you think there should be training on psychological skills to deal with patients’ anxiety, panic, and other emotional problems?	Yes	198	95.2%
	No	10	4.8%
8. Do you think the hospital should provide a place for rest where staff could temporarily isolate themselves from their family?	Yes	200	96.2%
	No	8	3.8%
9. Do you think staff should be available to deal with uncooperative patients?	Yes	206	99.0%
	No	2	1.0%
10. Do you think there should be proper training regarding how to use protective equipment?	Yes	208	100.0%
	No	0	0.0%
11. Do you think maintaining the mental health of staff is essential to deal with patients?	Yes	208	100.0%
	No	0	0.0%
12. Do you think stress-relieving activities should be arranged for staff?	Yes	202	97.1%
	No	6	2.9%
13. Do you think psychological counselors should regularly visit to counsel staff at work and provide support if needed?	Yes	194	93.3%
	No	14	6.7%
14. Do you think your anxiety is affecting the quality of care patients should receive?	Yes	140	67.3%
	No	68	32.7%
15. Do you think working under these conditions would lead to residual long-term effects on your mental health?	Yes	145	69.7%
	No	63	30.3%
16. Do you feel overwhelmed and overextended with the workload?	Yes	160	76.9%
	No	48	23.1%
17. Do you have a history of physical and/or psychological disorders?	Yes	26	12.5%
	No	182	87.5%
18. Did you have any simultaneous exposure to other traumas or recent stressful situations?	Yes	36	17.3%
	No	172	82.7%
19. Do you experience unfavorable living conditions at the healthcare facility?	Yes	46	22.1%
	No	162	77.9%
20. Do you have a system of work rotation at your hospital?	Yes	137	65.9%
	No	71	34.1%
21. Do you take satisfactory precautionary measures?	Yes	124	59.6%
	No	84	40.4%
22. Do you have access to free counseling and therapy?	Yes	43	20.7%
	No	165	79.3%
23. Do you do any physical and relaxation exercises to relieve your stress?	Yes	35	16.8%
	No	173	83.2%
24. Do you get enough rest and sleep?	Yes	86	41.3%
	No	122	58.7%
25. Do you eat a regular balanced meal?	Yes	77	37.0%
	No	131	63.0%
26. Do you participate in family and social activities?	Yes	122	58.7%
	No	86	41.3%
27. Is lack of PPE one of the reasons for your reluctance?	Yes	161	92.5%^a^
	No	13	7.5%^a^
28. Is the fear of self-infection one of the reasons for your reluctance?	Yes	73	42.0%^a^
	No	101	58.0%^a^
29. Is the fear of transmitting the infection to family members one of the reasons for your reluctance?	Yes	129	74.1%^a^
	No	45	25.9%^a^
30. Is too much workload one of the reasons for your reluctance?	Yes	13	7.5%^a^
	No	161	92.5%^a^

Mental health concerns

All of the respondents (n=208) felt that the mental health of the staff was equally important to control the situation and to deal with patients. Of note, 67% of respondents (n=140) thought that their anxiety is affecting the quality of care patients should receive; 97% (n=202) of respondents felt that stress-reducing activities should be arranged for staff in this regard, and 93% (n=194) of the respondents reported that psychological counselors should be available to provide regular counseling and emotional support. Only 20.7% (n=43) of the physicians had access to free counseling.

Of the participants, 69% (n=145) felt that working in these circumstances would lead to some residual long-term effects on their mental health. And 76% (n=160) of respondents felt overwhelmed and overextended with the workload; 17% (n=36) of the respondents had simultaneous exposure to traumas and other stressful situations, and 22% of the participants (n=46) experienced unfavorable living conditions. Also, 65% of respondents (n=137) reported that they had a system of work rotation in their hospital.

Of note, 59% (n=124) of respondents stated that they took satisfactory precautionary measures; 16% (n=35) of respondents did physical and relaxation exercises to relieve stress. Only 37% of respondents (n=77) were able to eat a regular balanced meal. More than half of the respondents (58.7%, n=122) participated in family and social events regularly, and 58% of the participants (n=122) had not been able to get enough rest and were sleep-deprived.

Factors associated with the reluctance to treat COVID-19 patients

All 46 of the participants with unfavorable living conditions were reluctant to treat COVID-19 patients (p<0.001). Of the 71 participants who did not have a system of work rotation at their hospital, 61 were reluctant to treat COVID-19 patients (p=0.010).

Notably, 88% (n=145) of the 164 participants having elderly relatives at home were reluctant to treat COVID-19 patients (p=0.001). Among participants with no prior experience of working in an epidemic, 83.21% (n=119) were reluctant to treat COVID-19 patients (p=0.487); 81% (n=152) of the participants who received no prior training to deal with COVID-19 patients were reluctant to treat them (p=0.016). Of the 192 respondents who reported unavailability of masks and PPE, 86.45% (n=166) were reluctant to treat COVID-19 patients (p=0.01) (Table 3).

**Table 3:** Associations of questionnaire responses with reluctance to treat COVID-19 patients *Significant at <0.05 level. **Significant at <0.01 level COVID-19: coronavirus disease 2019

		Are you reluctant to treat COVID-19 patients?		P-Value
		Yes	No	
1. Are there people older than 60 years in your home that you are in contact with?	Yes	145	19	0.001**
	No	29	15	
2. Did you face a shortage of masks and other protective equipment?	Yes	166	26	0.01*
	No	8	8	
3. Do you experience an unfavorable living condition at the healthcare facility?	Yes	46	0	0.000**
	No	128	34	
4. Have you undergone a simultaneous exposure to other traumas or recent stressful situations?	Yes	36	0	0.001**
	No	138	34	
5. Do you think your anxiety is affecting the quality of care patients should receive?	Yes	122	18	0.042*
	No	52	16	
6. Do you know how to deal with patients unwilling to be quarantined or take protective measures?	Yes	16	7	0.057
	No	158	27	
7. Did you have training on how to approach and deal with these patients?	Yes	22	0	0.016*
	No	152	34	

## Discussion

The COVID-19 pandemic has claimed the lives of not only the general public but also many healthcare workers and is continuing to do so. The deaths of many healthcare workers, including doctors, have occurred due to the coronavirus pandemic [7-9]. The toll on the mental health of healthcare workers has been equally severe. Doctors all around the world have been facing the difficult task of "rationing" medical supplies and facilities. They have had to decide which of the patients should receive treatment on a priority basis. The complexity of this situation was rightly summarized by a BBC article titled "the young doctors being asked to play god" [5]. This adverse situation has led to heart-breaking scenarios and even the suicide of a doctor who was actively involved in treating patients with COVID-19 [10].

The sheer psychological impact of this grave situation is severe enough to leave a lasting mark on a doctor’s mental health. Apart from this, it has also given rise to multiple concerns among physicians treating COVID-19 patients, many of whom are often reluctantly engaged in treating COVID-19 patients. It was observed in our study that most of the concerns arose because of insufficient protective measures the healthcare workers were provided with. Excessive working hours, the overwhelming nature of the work, and fear of self-infection were also the reasons behind these concerns. We have observed in our study that many of our physicians expressed their wish to have psychological counseling and training.

Notably, 83% of our 208 participants expressed their reluctance to treat COVID-19 patients. The reasons included lack of protective gear, fear of self-infection and infecting family members, and excessive workload. Many of the doctors faced unfavorable living conditions. They also had to deal with uncooperative patients. A large number of physicians felt that their mental health was also affecting the level of care the patients should receive.

The results of our study in Pakistani healthcare settings are similar to those observed globally. A study conducted among healthcare workers in China also showed that a considerable proportion of healthcare workers reported experiencing symptoms of depression, anxiety, insomnia, and psychological distress [11]. Another study conducted in Hong Kong reported a high prevalence of distress among healthcare workers and linked it to three factors: vulnerability, fear for self-health, and spread of the virus [12].

Healthcare workers around the world have also expressed their concerns about unknowingly transmitting the infection to family members, especially those who are elderly or those with chronic medical conditions [13]. Another qualitative study assessing the challenges of working in the scenario of an infectious evolutionary and extra workload faced by healthcare workers reported that healthcare workers felt they were "powerless to help their patients". They also feared getting infected themselves as well as infecting further people, including family and patients. Their relationships were also affected. They also found working with protective gear and heavy workload exhausting [14].

Our study was an attempt to assess the mental burden this pandemic has put on the healthcare workers. In light of our findings, we recommend that adequate psychological help and counseling should be provided to the healthcare workers. Being the front-line warriors against the pandemic, we cannot afford to lose them at the hands of this contagious disease, and nor should we let them deteriorate psychologically. Therefore, the provision of an appropriate amount of PPE should be ensured. The working schedule should allow doctors to take breaks and should ensure that they are not overworked. Steps should also be taken by the government and healthcare officials to ensure that such problems are better dealt with in case of a reoccurrence.

## Conclusions

COVID-19 pandemic has wreaked unprecedented havoc on our healthcare system as well as healthcare personnel. Many of the physicians have expressed fear and reluctance to treat patients due to a lack of PPE, fear of self-infection, and fear of transmitting the infection to family members. In addition, they also face drastic circumstances that have taken a toll on their mental health, which in turn can affect the quality of care that the patients receive. Therefore, it has been suggested that adequate psychological counseling should be made available to healthcare workers. Steps need to be taken to provide them with all the support they require in order to ensure a healthy working environment as well as to make sure that the patients receive state-of-the-art quality of care.
